# D-dimer as a Predictor of ICU Admission and Mortality in COVID-19 Patients: Insights From a Two-Year Retrospective Study From a Tertiary Care Center in South India

**DOI:** 10.7759/cureus.70682

**Published:** 2024-10-02

**Authors:** Immanuel Ratan Stephen, Febe Renjitha Suman, Jayalakshmi Balasubramanian, Sri Gayathri Shanmugam, Rajkumar Mani

**Affiliations:** 1 Pathology, Sri Ramachandra Institute of Higher Education and Research, Chennai, IND; 2 Internal Medicine, Sri Ramachandra Institute of Higher Education and Research, Chennai, IND

**Keywords:** covid-19, d-dimer, hypercoagulability, icu admission, mortality prediction, prognostic marker, retrospective comparative study, serial monitoring, south india, thrombosis

## Abstract

Introduction

Severe acute respiratory syndrome coronavirus 2 (SARS-CoV-2)-induced pneumonitis results in a prothrombotic and hypercoagulable state. Prognostic indicators are crucial for identifying patients at risk of complications. D-dimer, a degradation product of cross-linked fibrin, is a specific marker for thrombosis. Elevated D-dimer levels have been strongly correlated with poor prognosis and increased severity of illness in COVID-19 patients. Given D-dimer’s eight-hour half-life, periodic measurement is necessary to track disease progression. This study aimed to analyze and derive threshold and peak D-dimer values to predict outcomes in COVID-19 patients, comparing those treated in isolation wards to those requiring intensive care.

Methods

This two-year retrospective observational study included patients above 18 years with confirmed COVID-19. Patients were categorized into those treated in isolation wards and those admitted to the intensive care unit (ICU). Based on the outcome, they were further divided into survivors and non-survivors. Demographic and outcome-related data were collected from the hospital's laboratory information system. Serial D-dimer measurements were taken at eight time points. Statistical analysis was performed using the Mann-Whitney test for laboratory values and the chi-square test for demographic data. Receiver operating characteristic (ROC) curve analysis was utilized to derive critical D-dimer values. The area under the curve (AUC) was calculated for initial and peak D-dimer values.

Results

Of 2.149 patients with confirmed COVID-19, 811 (38%) presented with elevated D-dimer levels. ICU admission was required for 239 patients, either due to direct admission or worsening conditions. An initial D-dimer value of ≥0.93 mg/L FEU indicated the need for ICU admission, while a peak D-dimer value of 5.65 mg/L FEU predicted mortality. The AUC for the initial D-dimer was 0.60 (95% CI: 0.55-0.64), indicating moderate discriminatory power. The AUC for the peak D-dimer was 0.58 (95% CI: 0.54-0.62), suggesting lower predictive accuracy for peak values. Sensitivity was high for both initial (0.925) and peak (0.960) D-dimer values, although specificity was lower, especially for the peak D-dimer (0.486), resulting in a higher rate of false positives.

Among the ICU patients, the age range was 27-97 years, with a mean of 53.5 years. Males were more affected than females (71% vs. 29%), with a male-to-female ratio of 1.4:1. Of the ICU patients, 64.8% recovered, while 35.2% succumbed to the disease. Younger patients (mean age: 50.5 ± 12 years) recovered faster than older patients (mean age: 64 ± 16 years), with a significant difference in recovery time (p < 0.001). Gender did not significantly impact outcomes (p = 0.743). Survivors spent less time in the ICU (3-7 days) compared to non-survivors (4-14 days) (p = 0.041).

Conclusion

Serial D-dimer monitoring is essential for predicting outcomes and guiding treatment in COVID-19 patients. Initial and peak D-dimer values can help identify patients requiring intensive care and those at risk of mortality, allowing for timely interventions. D-dimer levels should be integrated into routine clinical assessments for managing COVID-19 patients.

## Introduction

The first case of the global pandemic caused by severe acute respiratory syndrome coronavirus 2 (SARS-CoV-2) was reported in Wuhan, China, in December 2019 [1]. As of October 1, 2024, there have been 776,281,230 confirmed cases globally, with 7,065,880 deaths. India alone has reported 45,043,227 confirmed cases, according to the WHO[2]. The virus’s mutations, diverse clinical presentations, and variability in infectivity highlight the need for accurate triage assessment and thorough counseling of patients and their families regarding the anticipated course of treatment.

Pro-inflammatory cytokines, including IL-1, IL-2, IL-6, TNF-α, IFN-γ, IP-10, GM-CSF, MCP-1, and IL-10, are released during the course of infection. Some of these correlate with disease severity, contributing to the cytokine storm that can cause complications such as coagulopathy and acute respiratory distress syndrome (ARDS) [3]. D-dimer, a degradation product of cross-linked fibrin, is a specific marker for thrombosis, reflecting clot formation and subsequent fibrinolysis. Elevated D-dimer levels have been closely associated with adverse outcomes in COVID-19 patients.

Patients with severe disease require rapid triage and intensive management. In this study, we aimed to examine D-dimer levels at admission, monitor trends through serial measurements, and identify threshold and peak D-dimer values that predict mortality in critically ill patients. Given D-dimer's eight-hour half-life, serial monitoring is essential for accurate risk assessment [4].

## Materials and methods

Ethical clearance for this retrospective observational study was obtained from the Institutional Ethics Committee (for Medical PG Students) of Sri Ramachandra Institute of Higher Education and Research (SRIHER) Deemed to be University (DU) (approval no. CSP-MED/20/SEP/61/75) and the Indian Council of Medical Research. The study population included only adult patients (age >18 years) admitted to SRIHER, a tertiary care center in Chennai, India, with a positive RT-PCR for SARS-CoV-2 and concomitant findings on diagnostic imaging, over a two-year period.

Patients were categorized into three groups based on disease severity: 1) mild disease: patients with positive RT-PCR for SARS-CoV-2 and symptoms (fever, cough, sore throat, malaise, headache, muscle pain, nausea, vomiting, diarrhea, or loss of taste/smell) without dyspnea or lower respiratory disease (SpO_2_ ≥ 94% on room air); 2) moderate disease: patients with positive RT-PCR for SARS-CoV-2 and evidence of lower respiratory disease during clinical assessment or imaging, with SpO_2_ ≥ 94% on room air; and 3) severe disease: patients admitted directly to the ICU with dyspnea, SpO_2_ < 94% on room air, respiratory rate >30 breaths/min, or lung infiltrates >50%.

The population was further categorized as survivors and non-survivors. Demographic and outcome-related data were retrieved from the hospital information system. D-dimer assay levels were collected from the laboratory information system and measured using the particle-mediated immunoturbidimetric assay (INNOVANCE) on the SYSMEX CS-2400 coagulation analyzer. A normal D-dimer level was defined as <0.5 mg/L FEU.

Laboratory and clinical outcome data were entered and analyzed using Google Sheets (Google, California, USA) and IBM SPSS Statistics for Windows, version 26.0 (released 2019, IBM Corp., Armonk, NY). The Mann-Whitney test was utilized for statistical analysis of laboratory values, while the Chi-square test was employed for demographic data. A p-value of 0.05 or lower was considered significant. ROC curve analysis, with a 95% confidence interval, was performed to determine critical threshold and peak D-dimer values. The AUC was used to evaluate the discriminatory power of D-dimer levels.

## Results

Of the 2,149 patients with confirmed COVID-19, 811 (38%) presented with elevated D-dimer levels. Among these, 239 patients required ICU admission, including those directly admitted and those transferred from isolation due to worsening illness. An initial D-dimer of ≥0.93 mg/L FEU was associated with ICU admission, while a peak D-dimer of 5.65 mg/L FEU predicted an increased risk of mortality.

The age of ICU patients ranged from 27 to 97 years, with a mean of 53.5 years. A greater proportion of males (170, 71%) required ICU care compared to females (69, 29%), with a male-to-female ratio of 1.4:1. Of the ICU patients, 155 (64.8%) recovered, while 84 (35.2%) succumbed to the illness. Younger patients (mean age: 50.5 ± 12 years) recovered more quickly compared to older patients (mean age: 64 ± 16 years) (p-value < 0.001). Gender did not significantly impact outcomes (p-value = 0.743). Survivors spent less time in the ICU (3-7 days) compared to non-survivors (4-14 days) (p-value = 0.041).

The serial D-dimer values for survivors and non-survivors are presented in Table 1.

**Table 1:** Comparison of serial D-dimer values between survivors and non-survivors in patients requiring intensive care all D-dimer values are expressed in mg/L FEU as mean (± SD). FEU: fibrinogen equivalent unit, SD: standard deviation

Characteristic	D-dimer 1	D-dimer 2	D-dimer 3	D-dimer 4	D-dimer 5	D-dimer 6	D-dimer 7	D-dimer 8
Survivors	0.93 (±2.04)	1.47 (±3.59)	1.94 (±6.53)	2.18 (±7.44)	3 (±6.89)	2.88 (±6.62)	2.49 (±4.76)	2.12 (±7.23)
Non-survivor	1.5 (±4.27)	1.75 (±4.94)	3.83 (±10.06)	4.69 (±19.2)	5.78 (±18.99)	5.89 (±6.74)	6 (±8.81)	7.04 (±11.91
P-value	0.001	0.002	0.011	0.012	0.014	0.009	0.005	0.002

**Figure 1:**
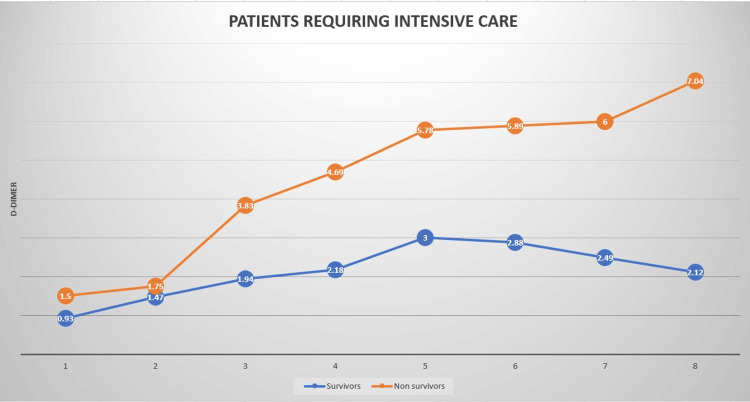
Dynamic changes in the D-dimer curve showing a significant rise among non-survivors as compared to survivors.

**Figure 2:**
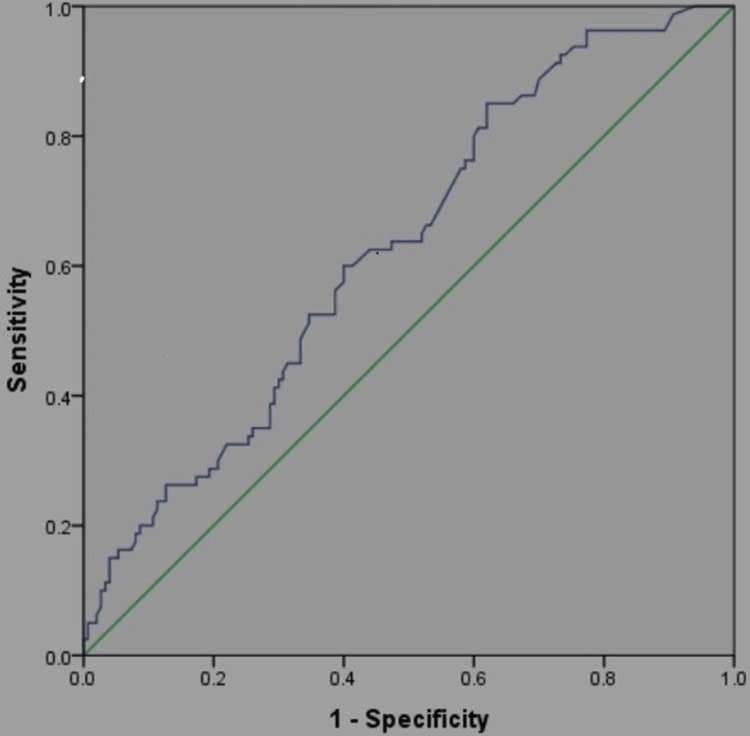
Receiver operator curve for the initial D-dimer.

**Figure 3:**
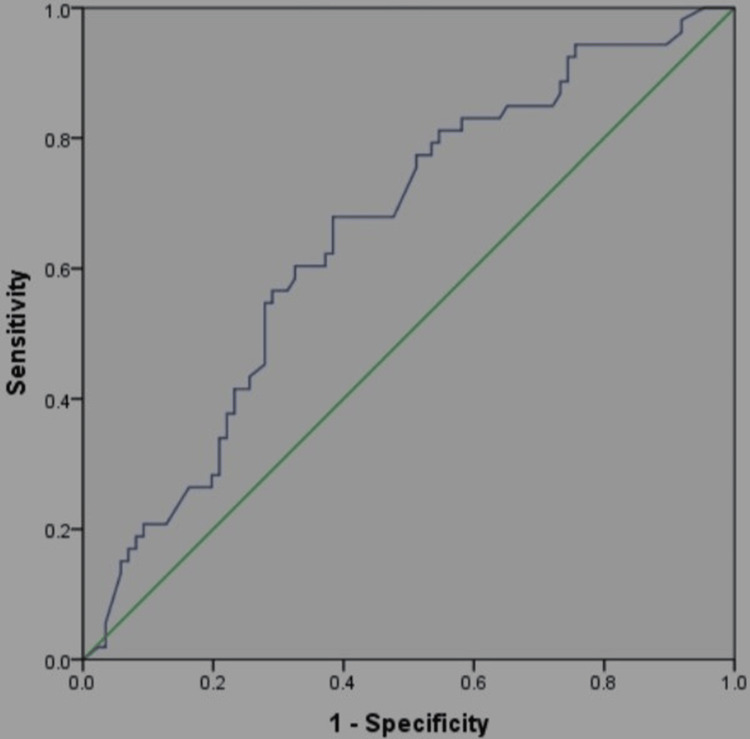
Receiver operator curve for the peak D-dimer.

There was a significant rise in D-dimer values among non-survivors in the later stages of the illness, while survivors exhibited relatively stable D-dimer levels, with a gradual decline (Figure 1). Figure 2 illustrates the receiver operating characteristic (ROC) curve used to derive the area under the curve (AUC) and cut-off values for initial D-dimer levels upon ICU admission. Figure 3 displays the ROC curve for peak D-dimer levels, which were used to determine the AUC and critical cut-off values for predicting outcomes in ICU patients. Table 2 presents the derived critical values for both the initial and peak D-dimer levels. 

**Table 2:** Area under the curve and derived critical cut-off values of D-dimer. AUC: area under the curve, CI: confidence interval

D-dimer	AUC (95% CI)	Sensitivity (95% CI)	Specificity (95% CI)	Positive predictive value (95% CI)	Negative predictive value (95% CI)
Initial	0.60 (0.55-0.64)	0.925 (0.844-0.972)	0.567 (0.498-0.606)	0.602 (0.431-0.747)	0.870( 0.737-0.951)
Peak	0.58 (0.54-0.62)	0.960 (0.863-0.995)	0.486 (0.335-0.513)	0.593 (0.506-0.771)	0.778 (0.400-0.972)

## Discussion

The AUC for both the initial and peak D-dimer values is relatively modest, with the initial D-dimer having a slightly better AUC (0.60) compared to the peak D-dimer (0.52). This suggests that D-dimer alone is not a perfect predictor of outcomes but may still have some predictive value. Sensitivity is high for both initial (0.925) and peak (0.960) D-dimer values, indicating that these measures are effective at identifying true positives (i.e., patients at risk). Specificity is relatively low, especially for the peak D-dimer (0.486), suggesting a higher rate of false positives. This means that high D-dimer values do not necessarily indicate poor prognosis for all patients. Both positive predictive values are below 0.5, indicating the limited ability of D-dimer to predict adverse outcomes. However, negative predictive values are higher (0.87 for initial and 0.778 for peak), suggesting that low D-dimer levels can reasonably predict better outcomes and a lower likelihood of severe complications.

The patients were categorized into two groups: those treated in isolation wards and those admitted to intensive care. Both groups had D-dimer values above the reference range (>0.5 mg/L). A significant portion of patients admitted to isolation wards had D-dimer levels below the reference cut-off and were included for comparative analysis. The age of patients ranged from 27 to 97 years, with a mean age of 53.5 years. Males were more affected than females in all groups, with a male-to-female ratio of 1.5:1. This age and gender distribution is consistent with other studies [5].

A comparative study was conducted between patients in the isolation ward with elevated D-dimer levels (n = 572, mean: 0.93 ± 0.37 mg/L FEU) and those with normal D-dimer levels (n = 1338, mean: 0.23 ± 0.16 mg/L FEU). Although there was a significant difference (p-value < 0.001), no adverse outcomes were documented in our hospital data (Table 3). This suggests that D-dimer cannot be considered an independent prognostic marker when only marginally elevated.

**Table 3:** Comparison of D-dimer (normal vs. elevated) among ward patients.

Number of patients	Mean ± SD (mg/L FEU)	P-value
572	0.93 ± 0.37	<0.001
1338	0.23 ± 0.16	

The initial mean D-dimer of survivors in the ICU (n = 154, mean: 2.08 ± 1.15 mg/L FEU) was significantly higher (p-value < 0.0001) compared to the initial mean D-dimer of patients in isolation wards (n = 572, mean: 0.93 ± 0.37 mg/L FEU) (Table 4). Although the difference was significant, both groups survived, indicating that a D-dimer level of <3 mg/L FEU cannot serve as an independent prognostic marker in COVID-19 disease.

**Table 4:** Comparison of D-dimer levels in survivors (isolation ward vs. intensive care)

Number of patients	Mean ± SD (mg/L FEU)	P-value
572	0.93 ± 0.37	<0.0001
154	2.08 ± 1.15	

There was a steady increase in D-dimer values among patients who were hospitalized for extended periods, regardless of the outcome. In our study, D-dimer levels were measured on eight consecutive days in ICU patients. The D-dimer values were significantly higher in non-survivors compared to survivors on all days except day 2 (Table 1).

An initial D-dimer value of ≥0.93 mg/L was a warning sign, and levels of ≥1.15 mg/L or higher indicated the need for ICU admission (Table 2). Peak D-dimer values of ≥5.65 mg/L FEU predicted mortality. These cut-offs were derived using ROC curve analysis, with a sensitivity and specificity of 60% and 58%, respectively.

The initial cut-off values for predicting mortality reported in these studies show variation (Table 5).

**Table 5:** Comparative analysis of various studies on initial and peak D-dimer in COVID-19 disease including the present study.

S. No.	Year of study	Location	Study type	Duration (days)	Participants (n)	Survivors (n)	Deceased (n)	Initial D-dimer (survivors)	Initial D-dimer (deceased)	p-value	Peak D-dimer (survivors)	Peak D-dimer (deceased)	p-value	Critical initial D-dimer	Critical peak D-dimer
1	2020	Wuhan, China [6]	Retrospective	76	349	297	52	0.35 mg/L (0.15–0.62)	1.81 mg/L (0.52–9.34)	<0.001	0.40 mg/L	29.44 mg/L	<0.001	0.73 mg/L	>3.78 mg/L
2	2020	Shanghai, China [7]	Retrospective	40	248	231	17	1.02 mg/L (0.47–2.62)	6.21 mg/L (3.79–16.01)	=0.00	-	-	-	>2.14 mg/L	-
3	2020	Chennai, India [8]	Retrospective	112	483	408	75	0.76 µg/mL	2.02 µg/mL	<0.01	0.945 µg/mL	6.34 µg/mL	<0.01	>1.44 µg/mL	>2.01 µg/mL
4	2020	Wuhan, China [9]	Retrospective	62	343	330	13	<2 µg/mL	>2.12 µg/mL	-	-	-	-	>2 µg/mL	-
5	2021	Kathmandu, Nepal [10]	Retrospective	270	182	145	34	1.067 µg/mL (±1.705)	3.208 µg/mL (±2.613)	-	-	-	-	>1.5 µg/mL	-
6	2022	Wuhan, China [5]	Retrospective	44	183	162	21	0.61 µg/mL (0.35–1.29)	2.12 µg/mL (0.77–5.27)	<0.01	-	-	-	-	-
7	2020	New York, USA [11]	Retrospective	31	375	215	160	831 ng/mL (408–2297)	394 ng/mL (268–677)	<0.0001	-	-	-	1000 ng/mL	-
8	2022	Trivandrum, India [12]	Retrospective	61	391	331	60	1.29 µg/mL (±2.08)	4.01 µg/mL (±3.53)	<0.001	1.50 µg/mL	5.73 µg/mL	<0.001	>1 µg/mL	-
9	2020-2022	Present Study	Retrospective	731	811	726	85	0.82 mg/L (±1.19)	1.5 mg/L (±4.27)	<0.01	2.12 mg/L	7.04 mg/L	-	>1.15 mg/L	5.65 mg/L

For instance, the cut-off in the Chinese study [6] was 0.73 mg/L, while in the Chennai, India study [8], it was 1.44 µg/mL, and in Kerala, India [12], it was >1 µg/mL. The present study derived a cut-off of 1.15 mg/L FEU, aligning closely with the values from these previous studies. Notably, a study conducted in Nepal reported higher initial D-dimer values among non-survivors, with a mean of 3.208 µg/mL, which was greater than the cut-off observed in our study and most others [5].

Peak D-dimer values were evaluated in only three studies, with significant variation. Among survivors, peak values ranged from 0.40 mg/L to 1.5 mg/L, while in non-survivors, values ranged from 5.73 mg/L to 29.44 mg/L. In our study, we identified a peak cut-off of 5.65 µg/mL to predict mortality. This is comparable to the cut-offs found in other studies, such as 3.78 mg/L (China) [6] and >2.01 µg/mL (Chennai, India) [8].

## Conclusions

The current study reinforces the utility of D-dimer as a critical biomarker in predicting the severity and outcomes in COVID-19 patients. Initial D-dimer levels of ≥0.93 mg/L FEU were associated with a higher likelihood of ICU admission, while a peak D-dimer value of ≥5.65 mg/L FEU was a strong predictor of mortality. These findings align with previously published studies from China, India, and Nepal, which reported similar critical cut-off values for predicting severe outcomes.

Our results also demonstrated that while the initial D-dimer levels offer moderate discriminatory ability with an AUC of 0.60, their high sensitivity (0.925) makes them a reliable tool for identifying at-risk patients. However, specificity was modest, particularly for peak D-dimer levels (0.486), indicating a higher likelihood of false positives. Nevertheless, the negative predictive value (NPV) of 0.87 for the initial D-dimer highlights the effectiveness of this marker in ruling out severe outcomes, providing reassurance for low-risk patients.

Comparisons with other studies showed similar critical D-dimer cut-offs, although our study's peak values were higher than those in several studies, suggesting that higher peak D-dimer levels may reflect more severe disease progression in our patient population.

Despite its utility, D-dimer levels should not be used as an independent prognostic marker, particularly when marginally elevated. Our study shows that patients with mildly elevated D-dimer levels did not experience adverse outcomes, even though their levels were statistically significant. Furthermore, D-dimer values below 3 mg/L FEU in survivors suggest that very high levels are more indicative of poor prognosis, particularly when monitored serially.

The serial monitoring of D-dimer in COVID-19 patients is critical for assessing disease progression and guiding treatment decisions. Initial D-dimer levels can serve as an early warning signal, especially for ICU admission, while peak values help predict mortality risk. This simple, cost-effective marker should be integrated into routine clinical evaluations, alongside other clinical and diagnostic parameters.

This study could not assess the impact of comorbidities, concurrent therapies, and vaccination status on D-dimer levels, which may have influenced the results. Further prospective studies are needed to explore these factors and their relationship with D-dimer trends in COVID-19 patients.

In conclusion, while D-dimer is a valuable marker in COVID-19, its interpretation should be context-dependent, considering other clinical indicators. Proper use of D-dimer monitoring can improve patient management, identify high-risk individuals, and prevent adverse outcomes through timely interventions.
